# Reliability of EEG Interactions Differs between Measures and Is Specific for Neurological Diseases

**DOI:** 10.3389/fnhum.2017.00350

**Published:** 2017-07-05

**Authors:** Yvonne Höller, Kevin Butz, Aljoscha Thomschewski, Elisabeth Schmid, Andreas Uhl, Arne C. Bathke, Georg Zimmermann, Santino O. Tomasi, Raffaele Nardone, Wolfgang Staffen, Peter Höller, Markus Leitinger, Julia Höfler, Gudrun Kalss, Alexandra C. Taylor, Giorgi Kuchukhidze, Eugen Trinka

**Affiliations:** ^1^Department of Neurology, Christian Doppler Medical Centre and Centre for Cognitive Neuroscience, Paracelsus Medical University SalzburgSalzburg, Austria; ^2^Spinal Cord Injury and Tissue Regeneration Center, Paracelsus Medical UniversitySalzburg, Austria; ^3^Department of Computer Sciences, Paris Lodron University of SalzburgSalzburg, Austria; ^4^Department of Mathematics, Paris Lodron University of SalzburgSalzburg, Austria; ^5^Department of Neurosurgery, Christian Doppler Medical Centre, Paracelsus Medical University SalzburgSalzburg, Austria; ^6^Department of Neurology, Franz Tappeiner HospitalMerano, Italy

**Keywords:** reliability, EEG connectivity, mild cognitive impairment, subjective cognitive complaints, temporal lobe epilepsy

## Abstract

Alterations of interaction (*connectivity*) of the EEG reflect pathological processes in patients with neurologic disorders. Nevertheless, it is questionable whether these patterns are reliable over time in different measures of interaction and whether this reliability of the measures is the same across different patient populations. In order to address this topic we examined 22 patients with mild cognitive impairment, five patients with subjective cognitive complaints, six patients with right-lateralized temporal lobe epilepsy, seven patients with left lateralized temporal lobe epilepsy, and 20 healthy controls. We calculated 14 measures of interaction from two EEG-recordings separated by 2 weeks. In order to characterize test-retest reliability, we correlated these measures for each group and compared the correlations between measures and between groups. We found that both measures of interaction as well as groups differed from each other in terms of reliability. The strongest correlation coefficients were found for spectrum, coherence, and full frequency directed transfer function (average rho > 0.9). In the delta (2–4 Hz) range, reliability was lower for mild cognitive impairment compared to healthy controls and left lateralized temporal lobe epilepsy. In the beta (13–30 Hz), gamma (31–80 Hz), and high gamma (81–125 Hz) frequency ranges we found decreased reliability in subjective cognitive complaints compared to mild cognitive impairment. In the gamma and high gamma range we found increased reliability in left lateralized temporal lobe epilepsy patients compared to healthy controls. Our results emphasize the importance of documenting reliability of measures of interaction, which may vary considerably between measures, but also between patient populations. We suggest that studies claiming clinical usefulness of measures of interaction should provide information on the reliability of the results. In addition, differences between patient groups in reliability of interactions in the EEG indicate the potential of reliability to serve as a new biomarker for pathological memory decline as well as for epilepsy. While the brain concert of information flow is generally variable, high reliability, and thus, low variability may reflect abnormal firing patterns.

## 1. Introduction

Interactions between neural signals are at the forefront of current neuroscientific research, which is also emphasized by the most recent name for this phenomenon: connectomics (Behrens and Sporns, 2012; Van Essen et al., 2012; Emmons, 2015; Sporns, 2015). The assessment of the connectome has attracted particularly great interest with regard to brain disorders (Fornito et al., 2015).

In mild cognitive impairment (MCI), interaction between EEG-signals (today most often known as connectivity Aertsen and Preissl, 1991) was found to be a reliable marker for cerebral reserve capacity (Teipel et al., 2016), response to interventions (Klados et al., 2016), and to monitor and predict disease progression from MCI to Alzheimer's disease (Rossini et al., 2006; Giannakopoulos et al., 2009; Drago et al., 2011; Dai and He, 2014; Hsiao et al., 2014; Wurtman, 2015; Babiloni et al., 2016; Vecchio et al., 2016).

Regarding epilepsy in particular, scientific interest in connectomics in general (Engel et al., 2013) and in neurophysiological interactions (Lehnertz et al., 2009) is clearly evidenced by the large number of studies conducted; a pubmed search for *connectivity epilepsy EEG*, yields 437 articles, whereas *connectivity mild cognitive impairment EEG* yields 92 (search performed on March 13th, 2017). The first measure of interaction, coherence (Walter, 1968), has been used to locate the epileptic focus as early as 1970 by Gersch and Goddard (1970). Newer approaches are centered on directed measures of interaction, which can be seen in the larger concept of Granger causality (Granger, 1969). Directed interaction is a statistical characterization of potential causality or information flow. As such, information sources and sinks can be identified, where sources are electrodes or other units of measurement which influence activity of other units, while sinks are units which are mainly influenced by other sources. These connectivity measures are still in use and have undergone further development (Korzeniewska et al., 2003), such as in a combination with wavelets (Li et al., 2007).

Today, in epilepsy research, directed measures are favored over undirected measures, because they can be used to model the spreading activation of ictal and interictal epileptic activity (Schevon et al., 2007; Lemieux et al., 2011; van Mierlo et al., 2011; Dai et al., 2012; Stefan and Lopes da Silva, 2013; Pittau et al., 2014; van Mierlo et al., 2014; Varotto et al., 2015).

Research tends to underestimate the fact that statistical measures for interactions in general cannot replace the assessment of structural connections (Horwitz, 2003; Rockland, 2015). Indeed, only a few studies have tried to link measures of interaction from the EEG to structural connectivity assessed by diffusion tensor imaging in MCI (Teipel et al., 2009; Garces et al., 2014; Vecchio et al., 2015). Additionally, volume conduction jeopardizes interpretability (Christodoulakis et al., 2015). Volume conduction and activity at the reference can lead to artificial high coherence values. Therefore, imaginary coherency (Nolte et al., 2004) and partial coherence (Gersch and Goddard, 1970) have been suggested. The subsequent development of directional measures, namely directed coherence (Saito and Harashima, 1981) and directed transfer function (Kaminskí and Blinowska, 1991) were combined with the concept of partial statistics in order to obtain partial directed coherence (Baccalá and Sameshima, 2001) and direct directed transfer function (Korzeniewska et al., 2003). Several variants of these measures have been developed, i.e., the full frequency directed transfer function (Korzeniewska et al., 2003) and the generalized partial directed coherence (Baccalá et al., 2007) which deal additionally with spectral and scaling characteristics of the signals. Another promising development is the combination of directed coupling with information theoretic approaches (Li and Ouyang, 2010; Liang et al., 2015).

A major problem with brain-network metrics is reproducibility (Welton et al., 2015). Despite the existence of statistical frameworks which have been validated as quantifying the stability of resting-state networks in magnetic resonance imaging (Bellec et al., 2010), such methods are not implemented as a standard procedure when studying interactions. Graph metrics from non-directed functional networks derived from magnetoencephalography yielded an average intraclass-correlation coefficient of 0.60–0.65 (Deuker et al., 2009; Jin et al., 2011). These are not convincingly high values; we therefore suggest that the assessment of reliability, in the sense of stability over time, is crucial for research on brain-networks.

Several factors affect reliability. First, in general, reliability is higher in lower frequency networks compared to beta- and gamma frequency ranges (Deuker et al., 2009; Jin et al., 2011; Kramer et al., 2011; Andellini et al., 2015; Miskovic and Keil, 2015). Second, reliability of networks are affected by the length of the time-series and the number of trials (Andellini et al., 2015; Miskovic and Keil, 2015). Third, signal-to-noise ratio plays an important role in reliability (Miskovic and Keil, 2015). Fourth, type of measure and the type of network characteristics exhibit varying reliability, since different measures might not necessarily respond in the same way to changes of brain state (Liang et al., 2016). Phase-dependent measures show lower reliability than absolute power and classical coherence over 30 days (Cannon et al., 2012). Long term follow-ups of up to 2 years revealed intra-class correlation coefficients of 0.68–0.80 for global interaction and of 0.12–0.73 for graph measures (Hardmeier et al., 2014). Fifth, most interestingly, disease-specific processes may affect stability of measured networks. Microstates of interaction patterns exhibit disease-specific alterations in patients with Alzheimer's disease (Hatz et al., 2015) and seizure-specific spatiotemporal features are stable for ictal functional networks (Martz et al., 2013), suggesting that the behavior of the networks over time contains significant clinical information.

However, it is often claimed that measures of interaction in terms of functional connectivity may aid surgical planning (e.g., Englot et al., 2015), and that they correlate with cognitive processes like memory (e.g., Watrous et al., 2013) despite there being no characterization of the reliability of the measured networks. When assessing interaction patterns as an indicator for the seizure onset zone, reliability is of high importance in order to provide reliable information for epilepsy surgery. Moreover, differences between patient groups may be inconsistent across studies due to poor reliability of measures of interactions. There is a need to quantify this inherent problem before transferring measures of interaction into clinical practice.

So far, reliability of non-directed or directed interactions and network characteristics was either assessed in healthy participants, or in patients, but the patterns of reliability were never compared across groups and measures. For the manyfold research approaches that compare interactions between patient populations, it is crucial that the reliability of the assessed measures is the same across groups. In contrast, we believe that such an assumption is highly likely to be violated. We hypothesize that there could be pathology-specific patterns of reliability of interaction.

In the present study, we compared the test-retest reliability of a set of interaction measures over two EEG recordings between patient populations. The chosen datasets were resting-EEG recordings in patients with MCI, subjective cognitive complaints (SCC), temporal lobe epilepsy (TLE), and healthy controls (HC).

## 2. Materials and methods

### 2.1. Ethics

This study was carried out in accordance with the recommendations of Good Clinical Practice, with written informed consent obtained from all subjects. All subjects gave written informed consent in accordance with the Declaration of Helsinki. The protocol was approved by the Ethics Commission Salzburg (Ethikkommission Land Salzburg; approval number 415-E/1429).

### 2.2. Subjects

We recruited a total sample of 70 participants at the Department of Neurology, Paracelsus Medical University Salzburg, Austria, from May 2012 to December 2015. After exclusion of participants who did not undergo both EEG-examinations (two TLEr, one TLEl, three HC) and whose EEG was of poor quality (one SCC, one TLEl, two HC) 60 participants remained for this analysis. Poor quality of the EEG was defined as less than 8 segments remaining after excluding segments according to the automatic data inspection (see Section 2.5). Table 1 gives an overview of the demographic characteristics of patients included in the subgroups. More detailed information can be retrieved from Table S1.

**Table 1:** Sample overview.

**Sample**	***N***	**Median age**	**Age range**	**N women**	**N right-handed**
MCI	22	68.5	48–76	11	21
SCC	5	57	52–74	2	5
TLEr	6	33.5	21–51	3	5
TLEl	7	55.0	36–66	6	7
HC	20	61.5	23–74	14	18

*N, number; MCI, mild cognitive impairment*.

*SCC, subjective cognitive complaints*.

*TLEr, right lateralized temporal lobe epilepsy*.

*TLEl, left lateralized temporal lobe epilepsy*.

*HC, healthy controls*.

Structural MRI was obtained from all participants at the day of the second EEG recording. Clinical evaluation, specifically of the hippocampi, was performed by a board certified neurologist (co-author Giorgi Kuchukhidze). The esults of this evaluation are given in Table S1.

Patients with amnestic MCI or SCC were recruited in the memory outpatient clinic of the Department of Neurology, Paracelsus Medical University Salzburg, Austria. We defined patients with amnestic MCI according to level three and patients with SCC according to level two of the global deterioration scale for aging and dementia described by Reisberg et al. (1982) and Gauthier et al. (2006). Diagnosis was based on multimodal neurological assessment, including imaging (high resolution 3T magnetic resonance tomography, and single photon emission computed tomography with Hexamethylpropylenaminooxim), and neuropsychological testing. Neuropsychological testing of these groups included the MMSE score (MCI median = 28.5, range 25–30; SCC median = 28.5, range 27–30). We excluded patients when inflammatory, vascular, metabolic, traumatic, or major depression, psychosis or any pharmacological therapy could better explain the memory impairment or the memory complaints.

Patients with refractory unilateral TLE were recruited in the epilepsy outpatient clinic of the Department of Neurology, Paracelsus Medical University Salzburg, Austria. Diagnosis was based on multimodal neurological assessment, including imaging (high resolution 3T magnetic resonance tomography, and single photon emission computed tomography with Hexamethylpropylenaminooxim), neuropsychological testing, and video-EEG examination for up to 5 days. We excluded patients with progressive lesions or immunological causes of epilepsy.

Table S2 provides patient characterization data for patients with TLE, including information about whether seizures occurred within 24 h before or after the EEG-recording took place (column “seizure”).

The sample of healthy participants was recruited among the students of the Paris Lodron University of Salzburg, Austria, as well as among senior citizens associations, in order to achieve a close resemblance to the age and sex of the patient groups. Healthy participants were free of a history of neurological or psychiatric diseases and were not receiving any psychoactive medication.

### 2.3. Pathological aspects

Table S3 lists self-reported medication of all participants in this study, Table S4 lists the results of the assessment of pathological findings and signs of sleepiness in the EEG by board certified neurophysiologists (column “findings”).

### 2.4. Data registration

EEG was recorded in a quiet room. Participants were instructed to stay awake with eyes closed. Recordings lasted for 2–3 min. We used a BrainCap with a 10–20 system and a BrainAmp (Brain Products GmbH, Germany) 16-bit ADC amplifier. The sampling rate was 500 Hz. Of the 32 recorded channels, one was used to monitor the lower vertical electrooculogram and one was used to measure electrocardiographic activity. Two were positioned at the earlobes for re-referencing purposes to remove the bias of the original reference, which was placed at FCz. Data analysis was conducted for data collected from the remaining 27 electrodes F3, F4, C3, C4, P3, P4, O1, O2, F7, F8, T7, T8, P7, P8, Fz, Cz, Pz, FC1, FC2, CP1, CP2, FC5, FC6, CP5, CP6, TP9, and TP10. Impedances were kept below 10 kΩ.

The two EEG sessions were arranged to take place at the same time of day. For most participants, EEG was performed within the same time-range around noon (1 p.m.). For most patients, we were able to arrange the recordings such that the time difference between the two recordings was less than 3 h. For three participants (HC, SCC, TLEl) the time difference was ~4 h, for two patients (MCI, TLEr) the time difference was 6 h, and for one HC the time difference was 11 h.

### 2.5. Data preparation

Data was pre-processed with Brain Vision Analyzer (Version 1.05.0005, Brain Products GmbH). In order to re-reference all channels, a new reference was built by averaging the signal of earlobe electrodes. Butterworth Zero Phase Filters were used for a high-pass filter from 1 Hz (time constant 0.1592 s, 48 dB/oct) and an additional notch filter (50 Hz) was applied.

An automatic artifact detection was carried out. Maximal allowed voltage step per sampling point was 50 μV (values which exceeded this threshold were excluded within a range of ±100 ms); maximal allowed absolute difference on an interval of 200 ms was 200 μV and lowest allowed absolute difference during an interval of 100 ms was 0.5 μV (values which exceeded this were marked with a surrounding of ±500 ms). The result of this artifact detection was reviewed visually. If data quality was poor due to noise on the reference electrodes, the dataset was excluded.

No artifact correction such as independent component analysis (ICA) was performed, since these methods of artifact removal can be problematic when assessing measures of interaction. The removal procedure is not unlikely to introduce artificial similarity between the signals. This is of course not recommendable for the present work, because it also increases the reliability, i.e., when a participant tends to have many artifacts because of an increased frequency of eye blinks or frequent movements, he will yield higher interaction between signals and a high reliability of measures of interaction if we correct the artifacts.

The preprocessed data was exported into a generic data format and imported to Matlab® (release R2010b, The Mathworks, Massachusetts, USA).

The data was then segmented into 500 ms segments (i.e., 250 sampling points) for each participant. If the segment overlapped with a marked artifact, it was excluded from further analysis for all channels. The purpose of this segmentation was to exclude the artifacts in a segment-wise manner, which allows a regular exclusion of data; i.e., when segments containing artifacts were excluded, they were always a multiple of 500 ms. Calculation of the measures of interaction was done on the continuous signal of the remaining concatenated segments.

**Figure 1:**
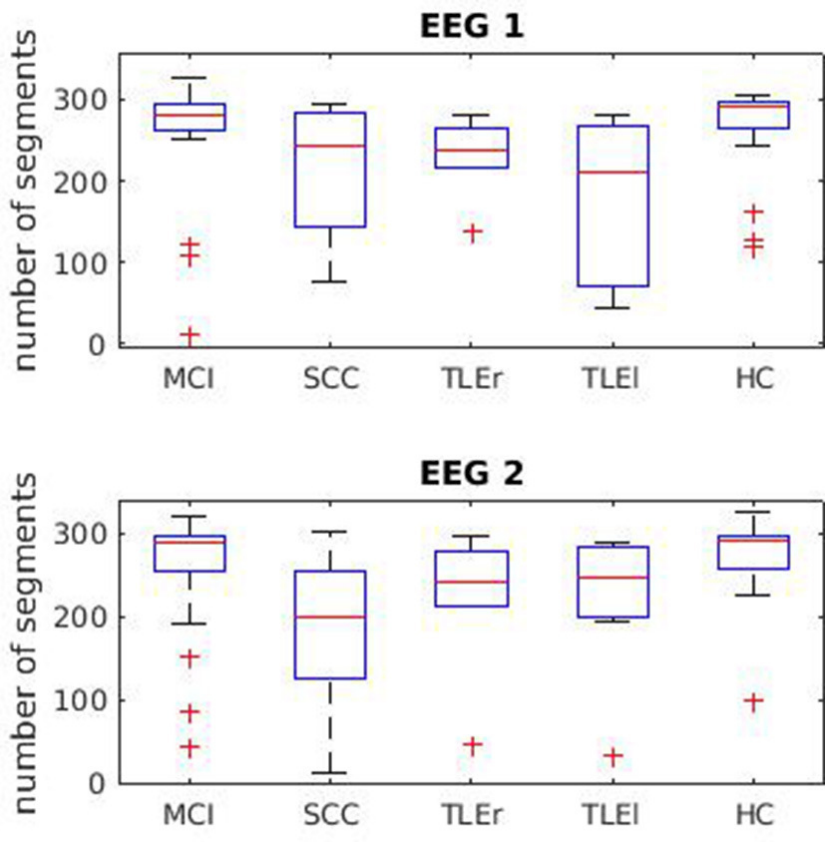
Boxplots for segment numbers. Segment (trial) numbers for the first and second EEG recording. One segment equals half a second. MCI, mild cognitive impairment; SCC, subjective cognitive complaints; TLEr, right lateralized temporal lobe epilepsy; TLEl, left lateralized temporal lobe epilepsy; HC, healthy controls.

Figure 1 shows the number of segments. One segment equals half a second. That is, for most participants we had at least 2 min of EEG for the analysis.

In order to estimate whether the reliability of the measures of interaction depends on the trial numbers that could be used for the calculation of the measures, we provide scatter plots in Figures S1–S14. The scatter plots show the relation between number of segments and test-retest reliability.

### 2.6. Feature extraction

We estimated a set of measures of interaction between all of the 27 selected electrodes (i.e., channels). The estimation was performed for each of the participants. The measures were calculated with the functions mvfreqz.m and mvar.m from the BioSig toolbox (Schlögl and Brunner, 2008) with model order 250 (i.e., equaling the length of the segmented data and enabling us to model at least one oscillation for each of the examined frequencies). To estimate the multivariate autoregressive model we used partial correlation estimation with unbiased covariance estimates (Marple, 1987), which was found to be the most accurate estimation method according to Schlögl (2006). The model is then transformed from the time-domain into the *z*-domain and the *f*-domain, which yields accordingly two transfer functions. The multivariate parameters in the frequency domain that can be derived from these transfer functions were computed for 1 Hz frequency steps between 2 and 125 Hz.

- **Spectrum:** This contains the auto- and the cross-spectrum, which is the Fourier transform of the cross-covariance function (Murthy, 1963).
- **Direct causality:** Direct causality was developed by Kaminski et al. (2001) to overcome the problem that the directed transfer function does not distinguish between direct and indirect information flows. Direct causality is the only measure that is not computed for each frequency.
- **Transfer function:** This transfer function is related to the non-normalized directed transfer function (Eichler, 2006).
- **Transfer function polynomial:** This is the frequency transform of a polynomial describing the transfer function. The absolute of the squared transfer function polynomial is the non-normalized partial directed coherence (Eichler, 2006).
- **Real valued coherence:** By considering the real part of the complex-valued coherence (Nolte et al., 2004), the result is an ordinary coherence (Schlögl and Brunner, 2008). We will refer to it as coherence.
- **Complex coherence:** By considering the imaginary part of the complex-valued coherence (Nolte et al., 2004), we get complex coherence.
- **Partial coherence:** This is the partial coherence, calculated with an alternative method as provided in the biosig-toolbox. Partial coherence, also known as Gersch causality, was first designed to identify epileptic foci by Gersch and Goddard (1970). The authors proposed that one channel is said to drive the other channels if the first channel explains or accounts for the linear relation between the other two. The real part of the partial coherence was used.
- **Partial directed coherence:** Partial directed coherence as an extended concept of partialized coherence, is a measure of the relative strength of the direct interaction between pairs of regions (Baccalá and Sameshima, 2001).
- **Partial directed coherence factor:** The partial directed coherence factor (Baccalá and Sameshima, 2001) is an intermediate step between partial coherence and partial directed coherence. It adds directionality to partial coherence, but includes instantaneous causality, which is undesirable when examining processes that evolve over time, such as an epileptic seizure (Schuster and Kalliauer, 2009).
- **Generalized partial directed coherence:** The major advantage of generalized partial directed coherence (Baccalá et al., 2007) over partial directed coherence is its robustness against scaling differences between the signals (Taxidis et al., 2010).
- **Directed transfer function:** Like directed coherence, the directed transfer function represents information that flows from one region to another over many possible alternative pathways (Kaminskí and Blinowska, 1991).
- **Direct directed transfer function:** The direct directed transfer function extends the concept of the directed transfer function by distinguishing between direct and indirect causal relations of signals (Korzeniewska et al., 2003). As such, the concepts of partial coherence and the directed transfer function are combined.
- **full frequency directed transfer function:** The difference between the directed transfer function and the full frequency directed transfer function (Korzeniewska et al., 2003) is that the directed transfer function is normalized by the total frequency content of the considered frequency band, while the full frequency directed transfer function is normalized with respect to all the frequencies in the predefined frequency interval. As such, the full frequency directed transfer function prioritizes those frequencies which contribute the most to the power of the signal (van Mierlo et al., 2011).
- **Geweke's Granger Causality:** This is a modified version of Geweke's Granger Causality (Geweke, 1982), concretely the bivariate version as in Bressler et al. (2007).

Before statistically determining and evaluating the reliability of the measures of interaction, we averaged them in classical frequency ranges delta (2–4 Hz), theta (5–7 Hz), alpha (8–13 Hz), beta (14–30 Hz), gamma (31–80 Hz), and high gamma (81–125 Hz).

### 2.7. Statistical analysis

#### 2.7.1. Measuring test-retest reliability

We decided not to use the parametric intra-class-correlation to measure the test-retest reliability, but to perform a non-parametric Spearman correlation (like in Fein et al., 1983; Gasser et al., 1985; Salinsky et al., 1991) because we did not want to impose a model assuming a linear relation between measurements. For the intra-class correlation coefficient, negative correlation coefficients are often set to zero because a negative correlation would indicate no accordance between the two recordings, just like zero correlation. However, with the Spearman correlation the indices were rather very close to zero or positive, so that setting negative coefficients to zero had no effect on the results. Therefore, we did not change the coefficients.

We measured reliability by Spearman rank correlation between the two times of registration for each measure of interaction and for each of the 60 participants, across the Cartesian product of all frequency × electrode × electrode combinations (or electrode × electrode combinations for direct causality). This Cartesian product is thus a concatenation of all values obtained when calculating measures of interaction, i.e., each electrode-electrode combination, and each frequency in one long vector. The correlation is thus done for two such vectors, one representing the network characteristics obtained during the first EEG recording, and the second one representing the network characteristic obtained during the second EEG recording.

#### 2.7.2. Reliability comparisons

We assessed the reliability between features with boxplots. For all subsequent analysis steps, we selected the set of features with the highest correlation coefficients from this step.

In order to compare reliability of the most reliable features between groups of participants (MCI, SCC, TLEr, TLEl, and HC), we used a non-parametric multivariate analysis of variance (package npmv for R version 3.0.2, The R Foundation for Statistical Computing, Vienna, Austria; underlying mathematics for the package described in Bathke et al., 2008, package implemented by Burchett and Ellis, 2013). The diagnoses (MCI, SCC, TLEr, TLEl, and HC) were the factor group and the 14 features were the repeated measures. A *p*-value of < 0.05 was considered to be significant.

#### 2.7.3. Analysis of regional group differences

We performed Spearman's rank correlation of the two time points across group members for each frequency × electrode × electrode combination, separately for each group, based on the single-patient estimations for the measures of interaction that yielded high reliability across all groups and that showed significant group differences. Thus, instead of concatenating all region combinations and frequencies such as in Section 2.7.1 in which we calculated reliability for each subject, we now looked at the single region combinations and features, and concatenated the values of the subjects. This resulted in correlation coefficients for each group and each frequency × electrode × electrode (and × frequency) combination.

We transformed the correlation coefficients into z-scores using Fisher's r-to-z transformation in order to compare these correlation coefficients between groups. Then, these z-scores were compared using formula 2.8.5 from Cohen and Cohen (1983), taking into account the number of participants in each group. Finally, we merged all *p*-values of all group comparisons and performed the Bonferroni-Holm correction. The critical *p*-value for statistical significance was 0.000001.

## 3. Results

### 3.1. Reliability comparisons between measures of interaction

**Figure 2:**
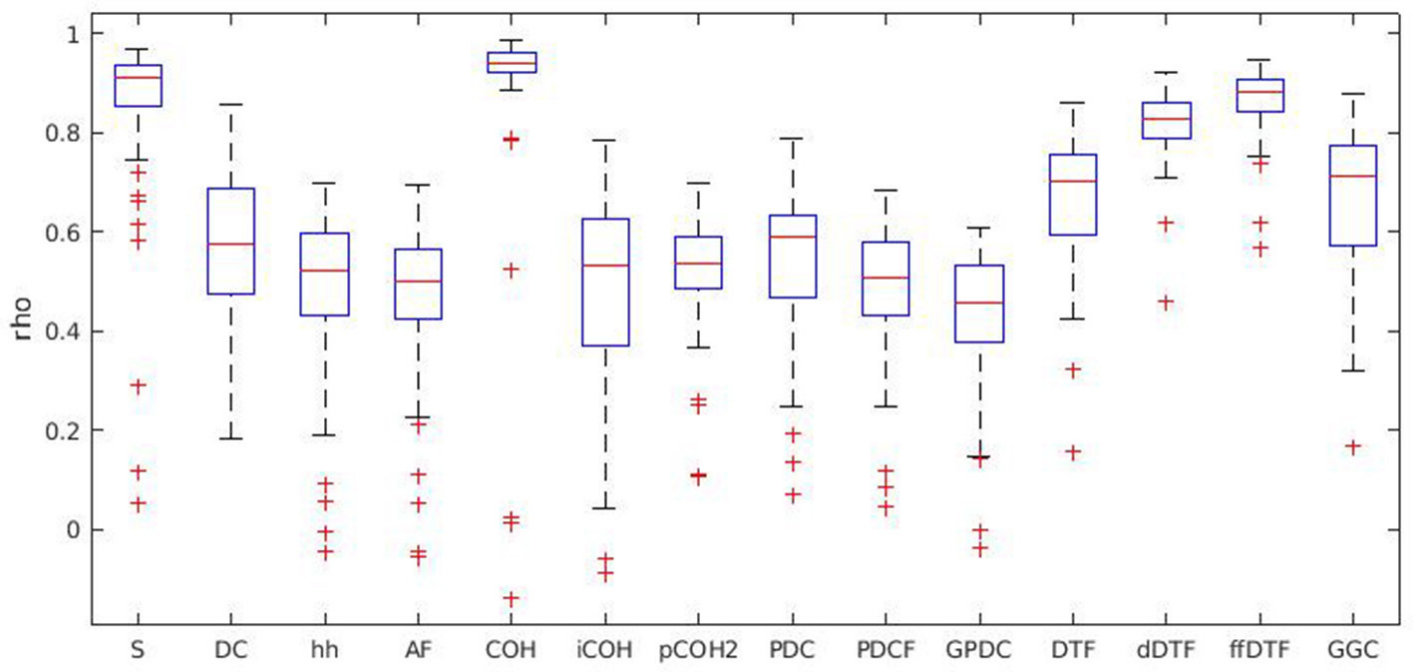
Boxplots for all measures of interaction. Boxplots show the distribution of Spearman's rho in the whole sample, individually for each measure. S, spectrum; DC, direct causality; h, transfer function; Af, transfer function polynomial; COH, real valued coherence; iCOH, complex coherence; pCOH, partial coherence; PDC, partial directed coherence; PDCF, partial directed coherence factor; GPDC, generalized partial directed coherence; DTF, directed transfer function; dDTF, direct directed transfer function; ffDTF, full frequency directed transfer function; GGC, Geweke's Granger causality.

Each measure's distribution of correlation coefficients across all participants is given in Figure 2. Each box is based on the distribution of the correlation coefficients rho (y-axis) of all participants, i.e., we have one rho for each participant.

Figure 2 suggests that the highest correlation coefficients were found for spectrum, real valued coherence, and full frequency directed transfer function. In that sense, these were the features with the highest reliability. Choosing these three features for further analysis allowed the consideration of one directional measure (full frequency directed transfer function), one non-directional measure without autocorrelation (real valued coherence), and one non-directional measure with autocorrelation (spectrum).

The scatterplots for trial numbers vs. test-retest reliability (Figures S1–S14) do not suggest a strong relation between trial numbers and reliability for these three measures. However, for other measures like partial coherence there is a trend toward lower reliability with lower trial numbers. For spectrum and real valued coherence the relationship is almost non-existent, while for full frequency directed transfer function a trend could be observed.

### 3.2. Reliability comparison between groups

The non-parametric multivariate ANOVA revealed a significant effect [*F*_(5.795, 54.066)_ = 2.519; *p* = 0.033]. The closed multiple testing procedure showed that the equality hypothesis for all possible quartetts of groups, and the direct comparison of the triplets (MCI, TLEl, TLEr), (MCI, SCC, TLEr), (MCI, SCC, TLEl), (HC, TLEl, TLEr), (HC, SCC, TLEr), (HC, MCI, SCC) and the pairwise comparison of MCI and SCC could be rejected.

**Figure 3:**
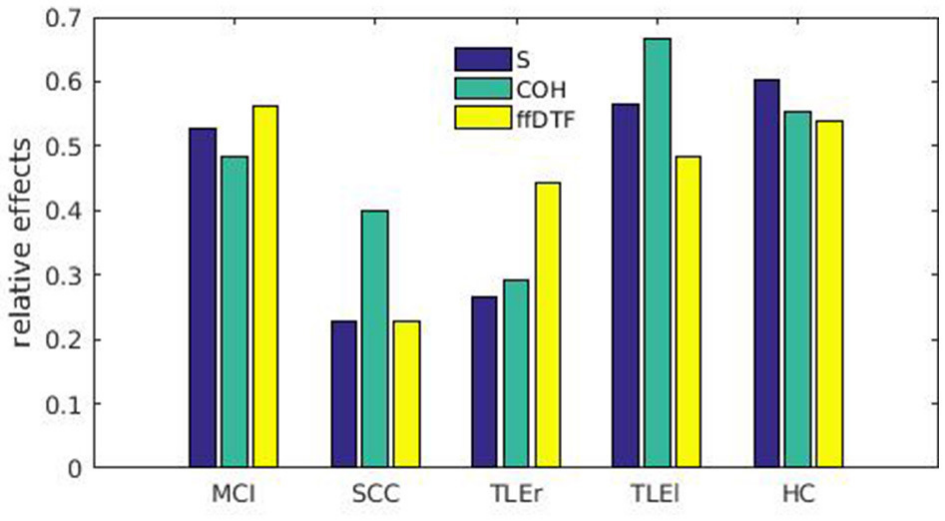
Relative effects for all measures of interaction sorted by groups. Relative effects are given for spectrum (blue), coherence (green), and full frequency directed transfer function (yellow). MCI, mild cognitive impairment; SCC, subjective cognitive complaints; TLEr, right lateralized temporal lobe epilepsy; TLEl, left lateralized temporal lobe epilepsy; HC, healthy controls. The relative effects indicate the probability that a randomly chosen participant from one group exhibits a larger reliability of a specific measure of interaction than a randomly chosen participant from all subgroups.

The relative effects are shown in Figure 3. The relative effects indicate the probability that a randomly chosen participant from one group exhibits a larger reliability of a specific measure of interaction than a randomly chosen participant from all subgroups. First of all, the relative effects vary considerably across measures of interactions for all groups, but not so much for MCI patients and HC. Patients with SCC seem to have the lowest probability of showing higher reliability of all groups. Patients with TLEr show lower probabilities than healthy controls, while there is not such a clear trend for TLEl and MCI.

### 3.3. Analysis of regional group differences

**Figure 4:**
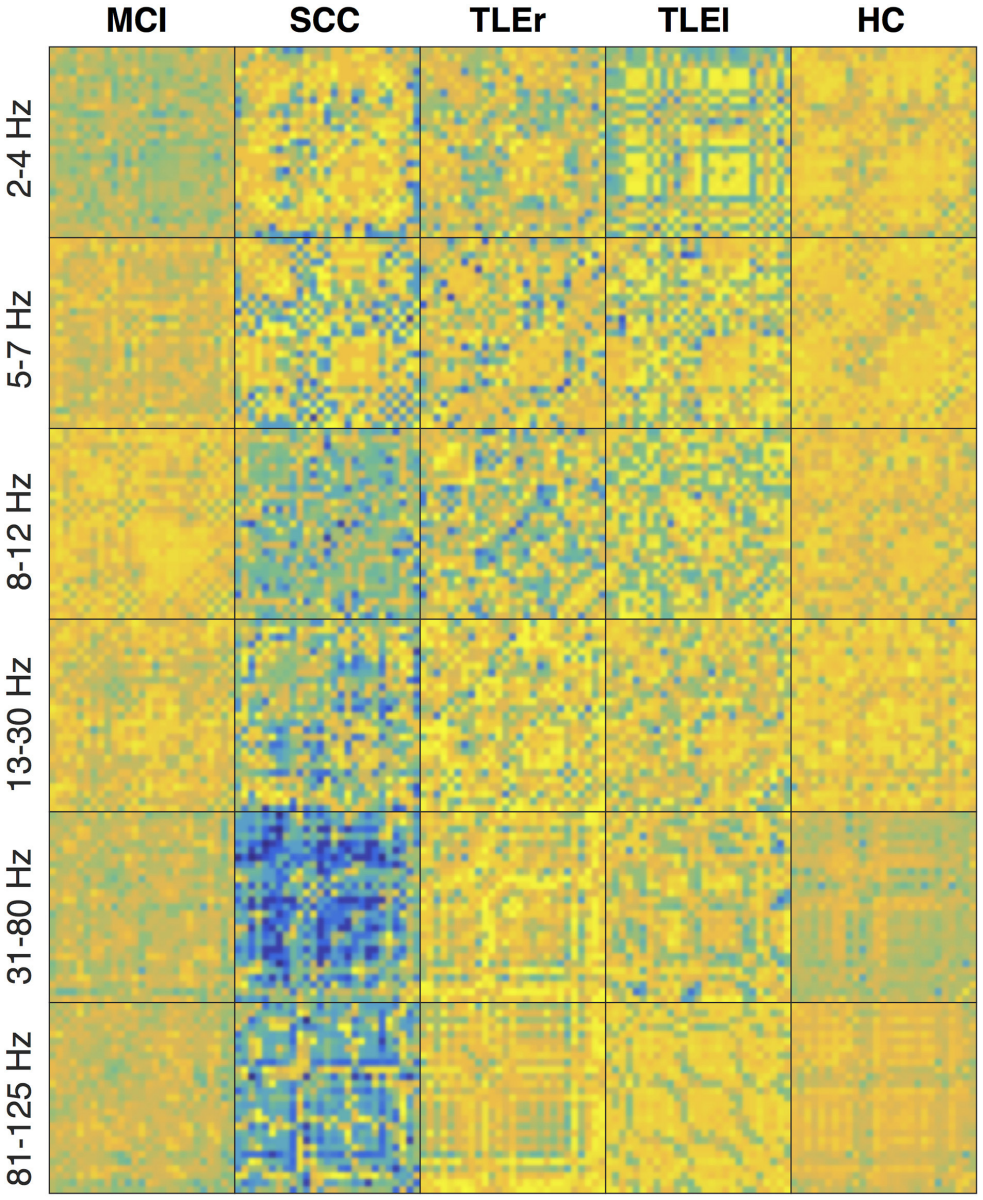
Heatmaps of the reliability of all electrode × electrode interactions for spectrum, sorted by frequency in rows and groups in columns. Colors indicate values from −1 (dark blue) to +1 (bright yellow). Electrodes start from top left following the order: F3, F4, C3, C4, P3, P4, O1, O2, F7, F8, T7, T8, P7, P8, Fz, Cz, Pz, FC1, FC2, CP1, CP2, FC5, FC6, CP5, CP6, TP9, and TP10. MCI, mild cognitive impairment; SCC, subjective cognitive complaints; TLEr, right lateralized temporal lobe epilepsy; TLEl, left lateralized temporal lobe epilepsy; HC, healthy controls.

**Figure 5:**
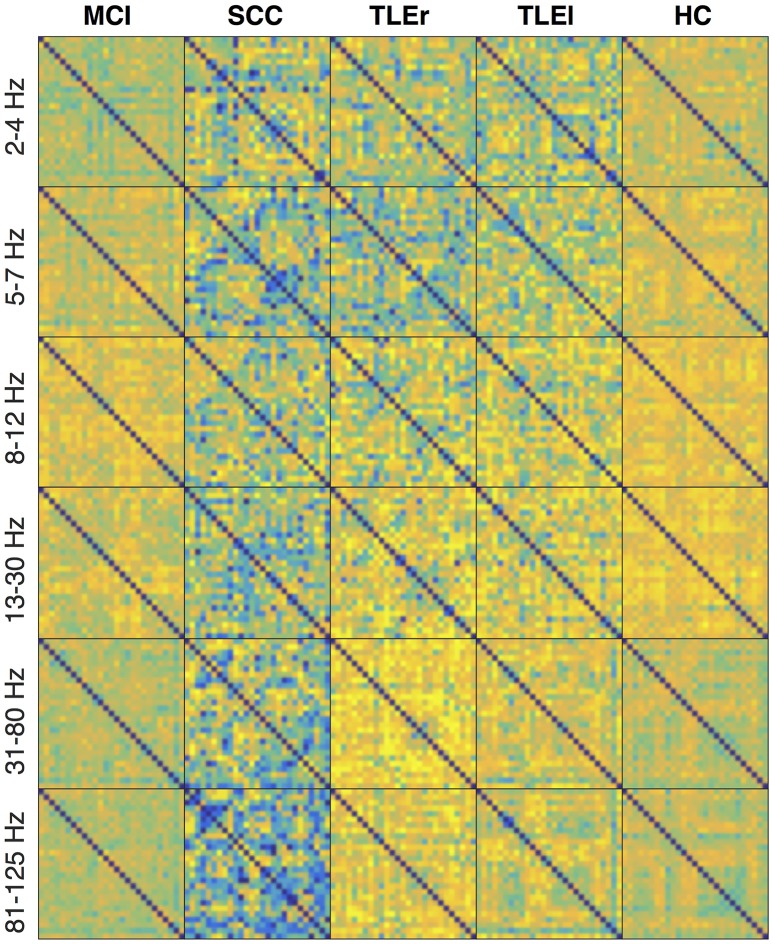
Heatmaps of the reliability of all electrode × electrode interactions for real valued coherence, sorted by frequency in rows and groups in columns. Colors indicate values from −1 (dark blue) to +1 (bright yellow). Electrodes start from top left following the order: F3, F4, C3, C4, P3, P4, O1, O2, F7, F8, T7, T8, P7, P8, Fz, Cz, Pz, FC1, FC2, CP1, CP2, FC5, FC6, CP5, CP6, TP9, and TP10. MCI, mild cognitive impairment; SCC, subjective cognitive complaints; TLEr, right lateralized temporal lobe epilepsy; TLEl, left lateralized temporal lobe epilepsy; HC, healthy controls.

**Figure 6:**
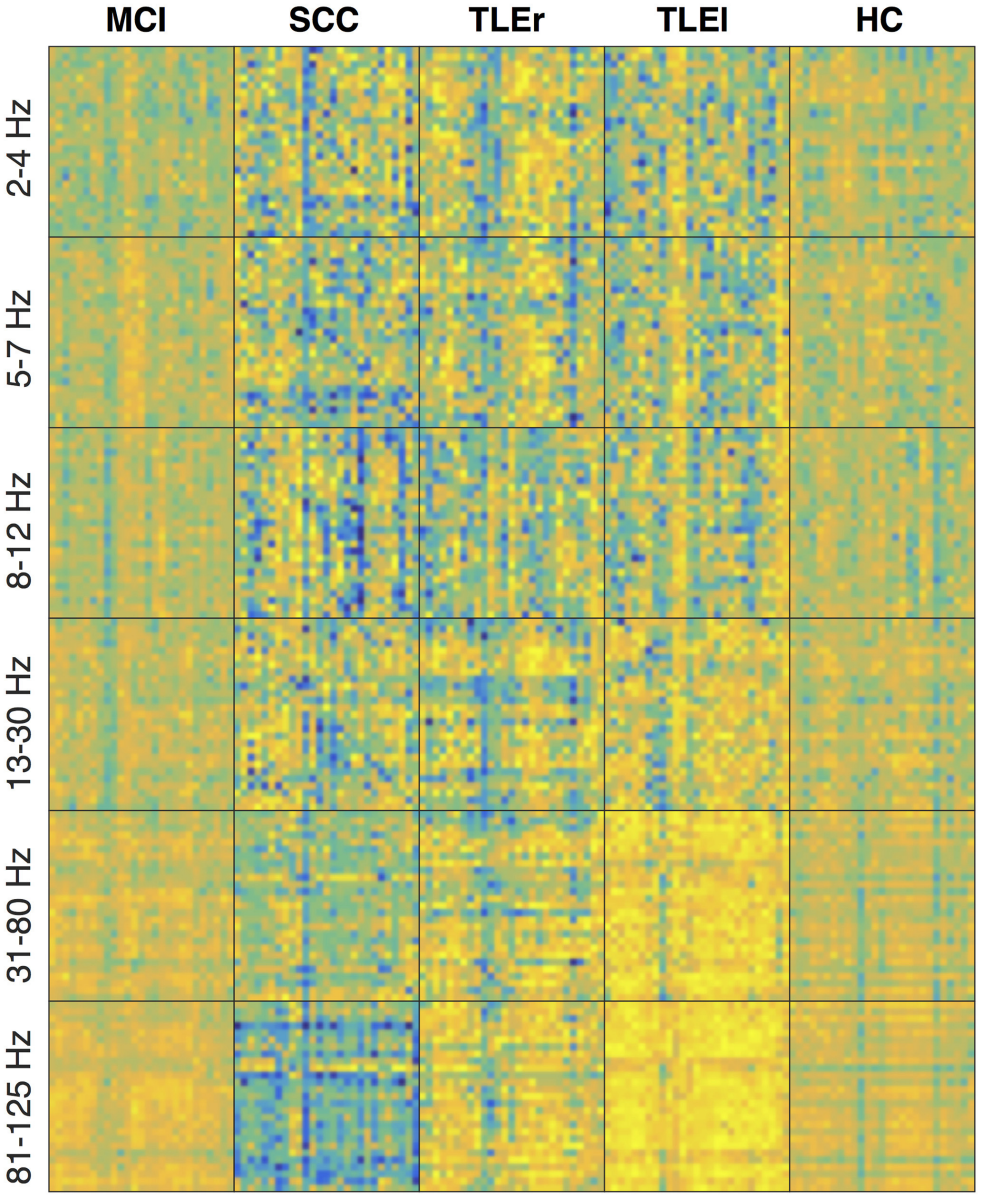
Heatmaps of the reliability of all electrode × electrode interactions for full frequency directed transfer function, sorted by frequency in rows and groups in columns. Colors indicate values from −1 (dark blue) to +1 (bright yellow). Electrodes start from top left following the order F3, F4, C3, C4, P3, P4, O1, O2, F7, F8, T7, T8, P7, P8, Fz, Cz, Pz, FC1, FC2, CP1, CP2, FC5, FC6, CP5, CP6, TP9, and TP10. MCI, mild cognitive impairment; SCC, subjective cognitive complaints; TLEr, right lateralized temporal lobe epilepsy; TLEl, left lateralized temporal lobe epilepsy; HC, healthy controls.

Figures 4–6 show the heatmaps of the reliability of all electrode × electrode interactions for spectrum, real valued coherence, and full frequency directed transfer function, respectively, arranged for frequencies and groups in rows and columns. A heatmap is a colorful representation of the network matrix. Each measure of interaction yields a matrix where each electrode represents one row and one column. The reliability of interaction is indicated as a colored dot for each electrode × electrode combination. Green and blue colors indicate low reliability, red colors indicate high reliability. Way easier to understand but less compact are topoplots, which can be found in the Supplementary section. Topoplots for spectrum, real valued coherence, and full frequency directed transfer function, and all frequencies are shown in Figures S15–S32.

The heatmaps for spectrum (Figure 4) suggest that the reliability is lowest for patients with SCC in the upper frequency ranges, and in the delta and gamma ranges, patients with MCI also show low reliability. For spectrum, the reliability in general is highest for HC, whereas regional variability is lowest for HC. A similar trend of high reliability in the HC group can also be observed for real valued coherence (Figure 5) in the alpha and beta range. Again, patients with SCC show lower reliability in general, represented by more blue dots. This trend is also visible in the heatmaps for full frequency directed transfer function (Figure 6). Here, the most impressive difference is the low reliability for patients with SCC in the high gamma range and also some regional very low reliability in this patient group in other frequencies. In addition, one can notice very high reliability for patients with TLEl in the gamma and high gamma range.

**Figure 7:**
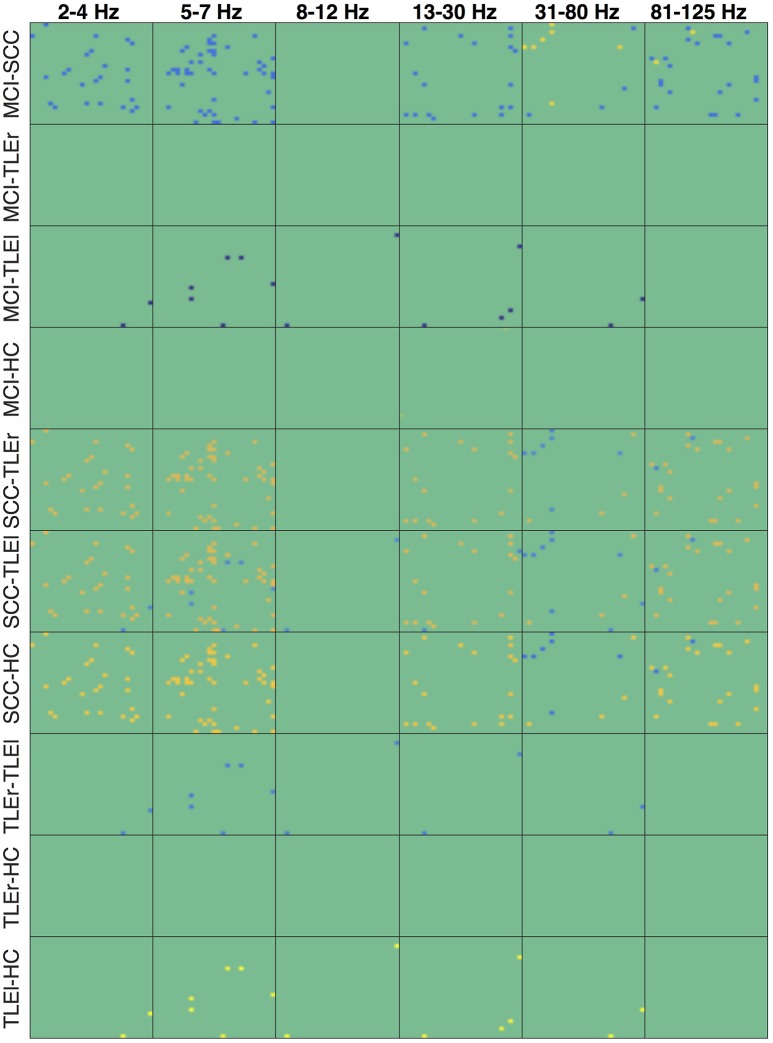
Heatmaps of group differences of the reliability for spectrum. Heatmaps show the reliability of all electrode × electrode interactions for measure of interaction spectrum, sorted by group comparisons in rows and frequency in columns. Each of the small boxes is drawn as the network matrix with one point corresponding to a specific electrode × electrode combination. Colors indicate values from −37.45 (dark blue) to +32.85 (bright yellow). Not-significant differences were set to 0 and appear in green. Electrodes start from top left following the order of the list as given in Section 2.4. MCI, mild cognitive impairment; SCC, subjective cognitive complaints; TLEr, right lateralized temporal lobe epilepsy; TLEl, left lateralized temporal lobe epilepsy; HC, healthy controls.

**Figure 8:**
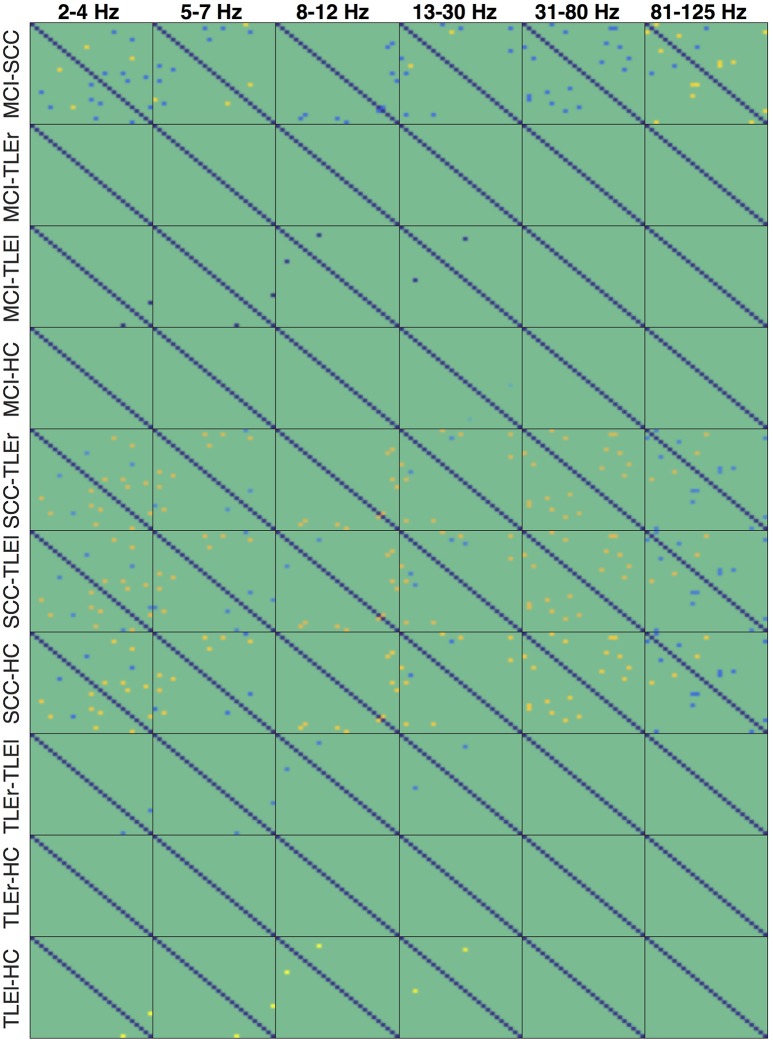
Heatmaps of the group differences in reliability for real valued coherence. Heatmaps show the reliability of all electrode × electrode interactions for measure of interaction real valued coherence, sorted by group comparisons in rows and frequency in columns. Each of the small boxes is drawn as the network matrix with one point corresponding to a specific electrode × electrode combination. Colors indicate values from −37.45 (dark blue) to +32.85 (bright yellow). Not-significant differences were set to 0 and appear in green. Electrodes start from top left following the order of the list as given in Section 2.4. MCI, mild cognitive impairment; SCC, subjective cognitive complaints; TLEr, right lateralized temporal lobe epilepsy; TLEl, left lateralized temporal lobe epilepsy; HC, healthy controls.

**Figure 9:**
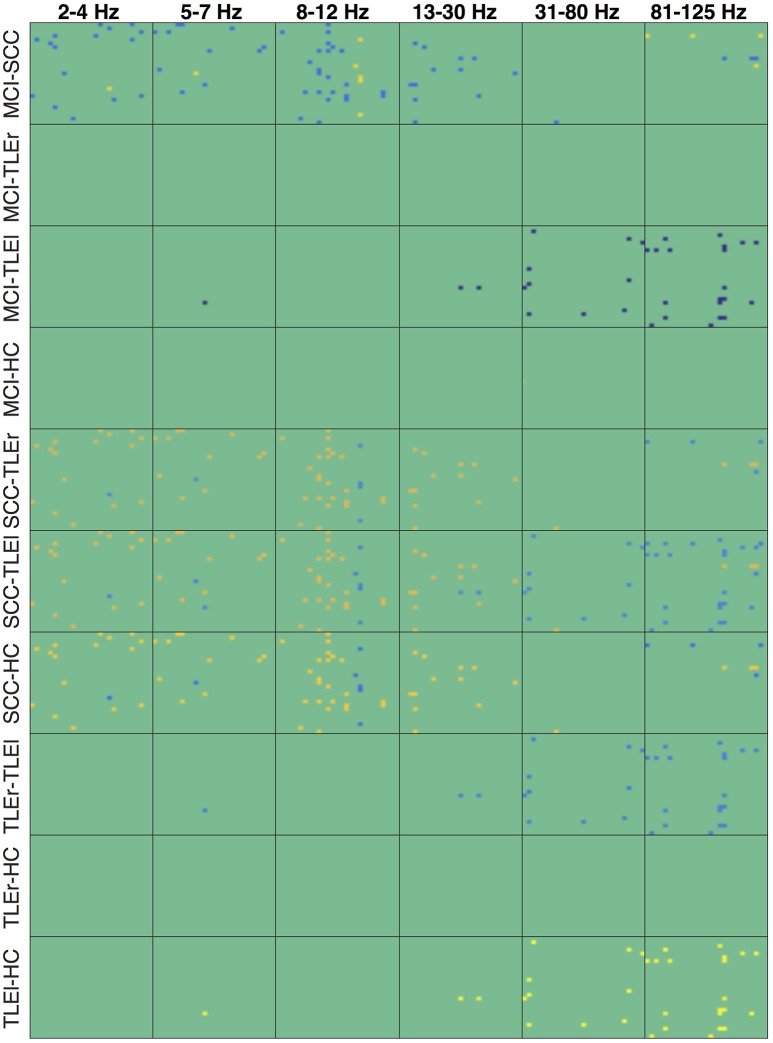
Heatmaps of the group differences in reliability for full frequency directed transfer function. Heatmaps show the reliability of all electrode × electrode interactions for measure of interaction full frequency directed transfer function, sorted by group comparisons in rows and frequency in columns. Each of the small boxes is drawn as the network matrix with one point corresponding to a specific electrode × electrode combination. Colors indicate values from −37.45 (dark blue) to +32.85 (bright yellow). Not-significant differences were set to 0 and appear in green. Electrodes start from top left following the order of the list as given in Section 2.4. MCI, mild cognitive impairment; SCC, subjective cognitive complaints; TLEr, right lateralized temporal lobe epilepsy; TLEl, left lateralized temporal lobe epilepsy; HC, healthy controls.

Heatmaps for statistically significant differences of group comparisons according to the r-to-z transform are shown in Figures 7–9 for spectrum, real valued coherence, and full frequency directed transfer function, respectively.

An notable difference is the significantly lower reliability of spectrum in all frequencies but alpha, for patients with MCI compared to SCC and TLEl. Figure S15 reveals that reduced reliability in the MCI group is widespread and that reliability is consistently high over almost all regions in HC. In contrast, TLEl patients show reduced delta reliability for interactions between other regions and the left temporal lobe. Another difference that is quite prominent is a higher reliability for patients with SCC compared to TLEr, TLEI, and HC groups in all frequency ranges but not in the alpha range, with regionally focused patterns. Topoplots in Figures S17–S19 suggest that, while patients with MCI exhibit reliability that is comparable to HC, patients with SCC show globally and focally reduced reliability. Figure S19 suggests that this reduced reliability can be focally attributed to left-parietal regions. Also of note are regionally confined spots of increased reliability in TLEl compared to HC.

For real valued coherence we find a similar pattern, however, this difference is regionally more restricted than in spectrum.

In the full frequency directed transfer function we found increased reliability in the gamma range and high-gamma range for patients with TLEl compared to other groups. Again, we find increased reliability in delta, theta, alpha, and low gamma range for SCC patients compared to other groups, with a small regional exception where reliability is lower. Figures S31, S32 immediately show the globally increased reliability in patients with TLEl in these frequency ranges and that patients with SCC show global and focal lower reliability than MCI patients.

## 4. Discussion

We found significantly different reliability between measures of interaction and—most importantly—between groups of participants. Our results emphasize that a pathology-specific pattern of network reliability should be taken into account in clinical studies involving measures of interaction.

### 4.1. Reliability of measures of interaction

Among 14 measures of interaction, reliability was higher for spectrum, real valued coherence and full frequency directed transfer function, where the latter was the most reliable measure that also showed only a few differences between patient groups. However, among these three measures, full frequency directed transfer function showed the strongest relation between trial numbers and test-retest reliability.

There are only a few publications considering the issue of reliability, at least as a by-product when studying measures of interaction. In recent studies (e.g., Schevon et al., 2007; Douw et al., 2010; Elisevich et al., 2011), it was shown that consistency was acceptable for a number of biomarkers in the EEG. The earliest measure of interaction that considered more than two channels was partial coherence, which was used for statistical partialization of correlations between triplets of electrodes by Gersch and Goddard (1970). The reliability of coherence has been examined in order to ascertain stability of findings. Elisevich et al. (2011) examined coherence of magnetoencephalographic signals in TLE patients in order to determine lateralization. Magnetoencephalographic coherence-based localization was evaluated against the standard single equivalent dipole model and postoperative outcome. In the work of Elisevich et al. (2011), reliability between runs was established by calculating the correlation between three consecutive epochs of 10 min each. The coherence analysis was not only more sensitive than the classical equivalent dipole model, but was also stable from run to run. However, the results obtained from magnetoencephalography may not be applicable to EEG. Specifically, coherences which are averaged over large frequency ranges (3–50 Hz) and several signals that form a collective source might be less variable than single-electrode coherences, so that the reliability of the averaged coherence is generally higher. In addition, the reliability of correlation values between consecutive recordings may be higher than in the case of recordings with more time in between them, e.g., 2 weeks, as in our study.

We would like to point out that coherence and newer measures of similarity between signals, e.g., the phase lag index and the frequency-entropy similarity measure by Gazit et al. (2011), are not able to disentangle the direction of information flow and could be hampered by volume conduction. It could be argued that there is lower variability for imaginary coherence because other methods are more sensitive to spurious connectivity due to volume conduction. Since volume conduction effects are not expected to change significantly between the two sessions, there will be less variability for the methods that are not robust against volume conduction effects, while imaginary coherence might mainly measure variability due to the physiological connectivity. If this argument were true, the reliability of physiological connectivity would be very low. However, our study compared directional and non-directional, direct and indirect measures of interaction. The superiority of the directed measures suggests that this characteristic may be of importance. This could also be explained by the fact that these measures can deal with the problem of volume conduction to some extent. However, we would like to note that none of the measures currently available can fully disentangle volume conduction from signal transmissions in the brain (Lehnertz, 2011). Directed measures are theorized to indicate Granger causality, which has gained popularity in neurosciences (Zhang et al., 2010). In particular in patients with epilepsy, directed measures have been shown to be useful in documenting the propagation of spikes (Lin et al., 2009) and to model evolving epileptic networks (Lehnertz et al., 2014). Accordingly, seizures were preceded by activities which were detectable intracranially by Granger causality in the high-frequency range (Adhikari et al., 2013). This is possible with a time and frequency domain Granger causality, realized by applying a sliding window (Lin et al., 2009; Adhikari et al., 2013), which is a common technique in event-related EEG. As a representative example, van Mierlo et al. (2011) used an adapted variant of the directed transfer function to document seizure onset and the ictal propagation pattern across several seizures.

However, the difference in reliability between the various measures of directed and non-directed interactions is considerable; we therefore suggest an amendment of checking for reliability whenever measures of interaction are subject to investigation.

### 4.2. Reliability differences between patient groups

We found a significant difference in reliability between patients with MCI, SCC, TLEr, TLEl, and HC. For the three selected measures of interaction, we found the highest reliability across all regions and frequencies for healthy controls. With respect to patients, there are focally and frequency-restricted alterations of reliability, which also depend on the measure of interaction. In the literature, reliability is higher in lower frequency networks compared to beta- and gamma frequency ranges (Deuker et al., 2009; Jin et al., 2011; Kramer et al., 2011; Andellini et al., 2015; Miskovic and Keil, 2015). However, this pattern seems to be specific for pathology. Thus, we need to direct our attention to frequency characteristics of reliability. Considering frequency and topography, we discuss here three very interesting patterns in the group comparisons:

First, our results on pathology-specific patterns of reliability oppose the typical findings for interactions. Previous research suggests increased interaction in lower frequencies and decreased interaction in higher frequencies as being associated with pathology in MCI and Alzheimer's dementia (Babiloni et al., 2016; Teipel et al., 2016). According to our results the interaction in lower frequencies was less reliable in patients with MCI, at least when comparing this group with patients with SCC. Interestingly, we found reduced delta reliability in patients with TLEl over the left side, while from literature we know that there might be focal hypersynchrony in TLE (Schevon et al., 2007). These results suggest that reliability and interaction values may exhibit opposite effects.

The second interesting finding was a variation of reliability in the gamma frequency ranges of SCC and MCI. While biomarkers from the EEG beta range are known to differentiate patients with stable from progressive MCI (Poil et al., 2013), the gamma range is not a typical frequency range of interest in dementia research. Instead, high frequency oscillations are both a potential marker for the delineation of the epileptogenic area in presurgical epilepsy patients (Worrell and Gotman, 2011; Jacobs et al., 2012; Staba et al., 2014; Höller et al., 2015) as well as a correlate of memory consolidation in the hippocampus (Axmacher et al., 2008; Buzsáki and Lopes da Silva, 2012; Mari et al., 2012); therefore there is a co-existence of pathological and physiological high frequency oscillations in patients with epilepsy. It would be natural to assume that disrupted memory occurs with reduced rates of high frequency oscillations in the temporal region. The present results suggest that the reliability in the frequency range where high frequency oscillations occur is decreased in SCC compared to MCI. However, these speculations need to be investigated with a different methodology. Studies on high frequency oscillations assess a distinct morphological pattern that can be identified either visually or automatically but that is so rare that it is not easily recognizable in the power spectrum. Moreover, reliability of interactions does not necessarily behave similarly to the strength of interaction. That is, the strength of interaction may be low, but this low interaction highly reliable over time; or a interaction may be high at instances of time, but is moderated by many factors so that it changes rapidly over time. Nevertheless, our results suggest that the examination of the high gamma range could be of interest in patients with SCC and MCI. Further research could examine the prognostic value of reliability of interactions for memory decline in these patient populations.

Third, reliability in the gamma and high-gamma range was increased for patients with TLEl compared to other groups. As described above, gamma and high-gamma activity is currently being discussed as a potential marker of epileptogenicity. Our results suggest that, in addition to the occurrence of high frequency oscillations, it could be worthwhile examining the reliability of the occurrence of these phenomena and the reliability of activity in the higher frequency range, in general. In support of this view, increased interactions were documented for the focal regions in patients with epilepsy. Schevon and her team Schevon et al. (2007) examined synchrony in the interictal intracranial EEG by calculating mean phase coherence. The focal hyperconnectivity was of a persistent spatiotemporal pattern, which was unique in each patient. Measures of interaction can also be a marker for epilepsy in brain tumor patients. Douw et al. (2009) found stable patterns of network topology over 6 months. Increased signal similarities according to phase lag index in the theta band were related to a higher number of epileptic seizures. With respect to our results, it is important to underline that the pathological alteration in Schevons and Douws data was reliable. The authors found consistent patterns both in three consecutive recordings of 5 min each as well as in five recordings, each separated by 1 day. Our findings suggest that reliability itself can provide important information.

However, it is not clear why this pattern could be found for the contrast between TLEl and HC but not for TLEr and HC. Speech dominance may explain this finding, but the present study does not include information on this for every patient. Another possible confounder could be the overrepresentation of women in the group of patients with TLEl. The menstrual cycle is known to affect the frequency of seizures (Herzog et al., 2015) and the EEG in general (Broetzner et al., 2014). It is possible that there is an effect of the menstrual cycle in women because of the study design, which has 2 weeks in between the two EEG recordings. However, we do not know how the menstrual cycle affects the reliability of measures of interactions. In addition, we would expect that fluctuations along the menstrual cycle would reduce reliability, whereas the group with an overrepresentation of women showed high reliability. The effect of the menstrual cycle on measures of interaction and their reliability needs to be considered carefully in future studies.

Finally, it is possible that the reasons for the varying reliability can be attributed straightforwardly to what can be extracted from the clinical EEG evaluation. The populations from which the study participants were drawn had nonspecific abnormalities (see Table S4) that could have affected results. It is not unlikely that the pathological patterns the clinicians evaluate qualitatively may explain the reduced reliability. However, any study performing an analysis of connectivity has to consider this aspect thoroughly, since sporadic pathologic patterns may affect the result.

### 4.3. Limitations

We would like to emphasize again that a higher reliability does not allow inferences to be made regarding a high or low interaction. Other publications reported a pathological or focal hyperconnectivity, as reviewed recently Panzica et al. (2013). Our results indicate that the pattern of interaction differs in reliability between patients with different pathologies and healthy participants.

We argue that this difference might provide a pathological explanation, since the scientific audience of this article could doubt that the reliability of interactions over recordings obtained at an interval of 2 weeks truly arises from the brain. Instead, it could be the result of volume conduction or the muscle activity from the musculus temporales. However, volume conduction or a muscle artifact would not explain why there would be differences in localization or between different patient populations. Therefore, we assume that the identified differences in reliability are of pathologic nature.

It is important to note that we did not control for menstrual cycle, sleepiness, alcohol intake the day before, and consumption of caffeine or tobacco. These aspects may affect the reliability of interactions in the EEG. Before claiming that measures of interaction could be useful for clinical examination, the effect of these factors on reliability has to be characterized.

Technical aspects should be considered when interpreting results in the high-gamma range. High frequency oscillations on scalp EEG are difficult to record (Worrell et al., 2012). The sensitivity of scalp EEG to power changes in this frequency range could be doubted, but recent work demonstrates that activity within this frequency range can be detected on the scalp (Zelmann et al., 2014). However, our results can only point toward importance of examination of the higher frequency ranges with respect to interactions and reliability of interaction. It is possible that high frequency oscillations yield more interesting results in terms of reliability, but with the presented results we can not derive any conclusion about this phenomenon.

We have to emphasize that the segment numbers might not affect the reliability of spectrum and real valued coherence, but they seem to affect the reliability of measures such as the full frequency directed transfer function. When looking at the scatterplots in the supplementary section, the relevance of data length becomes evident in a number of directed measures of interaction. This could of course bias the differences between the SCC patients and other groups, since SCC showed lower trial numbers than the other groups. The question as to why some measures are more sensitive to trial numbers than others has been addressed recently (Fraschini et al., 2016) and should be subject to future large studies involving direct comparison of more measures of interaction, and ideally also of different patient populations. Indeed, the short time duration (2–3 min) is typical of some studies, but longer time periods may have resulted in greater reliability and maybe less differences between groups. However, increasing the duration of recording resting EEG with eyes closed increases the probability that participants fall asleep. Effects of drowsiness might severely affect the reliability (Horovitz et al., 2008), especially when patients fall asleep at one session but not at the other session.

Next, the limited number of channels reduces the strength of the study. Specifically, the analysis was done only at sensor level and not at source level, also because the low number of electrodes would be a limitation to performing a proper analysis at the source level. Source-level connectivity analysis is also a promising approach (Schoffelen and Gross, 2009; Deligianni et al., 2014; Papadopoulou et al., 2015; Sockeel et al., 2016) because it is robust against volume conduction (Haufe et al., 2010; Drakesmith et al., 2013; Chella et al., 2016), and it yields clinically valuable information in epilepsy (Coito et al., 2015, 2016a,b; Hassan et al., 2017), MCI (López et al., 2014), and Alzheimer's disease (Canuet et al., 2012). Future work should determine whether interactions are more reliable at the source- than at the sensor level.

The group of TLE patients was considerably younger than the other groups, which might cause some bias. This is an unavoidable characteristic when comparing patients with prodromal dementia with patients with TLE; dementia occurs typically at an advanced age, while patients with TLE are typically younger. However, we did not find such marked ifferences with respect to reliability when comparing the youngest group (TLEr) to other groups, while the group of TLEl patients exhibited some differences. The TLEl group was also a bit younger compared to the other groups, but the most interesting speciality of this group was that it consisted mostly of women (6 out of 7 patients). With respect to a likely effect of menstrual cycle this could explain the reduced reliability in this patient group.

Finally, the patient numbers were low, especially in the TLE subgroups and in patients with subjective cognitive complaints. These groups, in turn, can be very heterogeneous in terms of medication and aetiology, which is often unknown. Especially in patients with subjective cognitive complaints we do not know whether this patient population suffers from a prodromal stage of dementia or whether they are just alerted to cognitive problems by the normal process of aging. It is desirable to merge international databases in order to assess reliability of measures of interaction on a large scale.

## 5. Conclusions and future directions

Our study suggests that reliability differs between measures of interaction and between patient populations.

Reliability of results over time is necessary both for replicability of scientific work, but even more when transferring knowledge about biomarkers from research into practice, specifically in pre-surgical evaluation or diagnosis and prognosis of dementia. The fact that the biomarker does not vary over time is detrimental for obtaining reliable information about the epileptogenic area and also for making therapeutic decisions due to an expected progression to dementia.

## Author contributions

YH performed the analysis and wrote the manuscript. AU, GZ, PH, and AB supervised the work in technical and statistical respects and contributed ideas to how the analysis should be performed and how the results should be presented. ACT, KB, and ES performed data acquisition. KB performed data preprocessing. ST, ACT, and GKu performed evaluation of structural images. ML, JH, and GKa performed recruitment and clinical evaluation of the EEG data. WS performed recruitment and evaluation of the patients with MCI. ET and RN supervised the work in clinical respects. All of the listed authors have read, commented and approved the manuscript.

### Conflict of interest statement

The authors declare that the research was conducted in the absence of any commercial or financial relationships that could be construed as a potential conflict of interest.
